# The Domain-Specific and Temperature-Dependent Protein Misfolding Phenotype of Variant Medium-Chain acyl-CoA Dehydrogenase

**DOI:** 10.1371/journal.pone.0093852

**Published:** 2014-04-09

**Authors:** Johanna M. Jank, Esther M. Maier, Dunja D. Reiß, Martin Haslbeck, Kristina F. Kemter, Marietta S. Truger, Christian P. Sommerhoff, Sacha Ferdinandusse, Ronald J. Wanders, Søren W. Gersting, Ania C. Muntau

**Affiliations:** 1 Department of Molecular Pediatrics, Dr. von Hauner Children’s Hospital, Ludwig-Maximilians-University, Munich, Germany; 2 Department of Chemistry, Technical University Munich, Garching, Germany; 3 Institute of Laboratory Medicine, Ludwig-Maximilians-University, Munich, Germany; 4 Departments of Laboratory Medicine and Pediatrics, Laboratory Genetic Metabolic Diseases, Academic Medical Center, Emma Children's Hospital, University of Amsterdam, Amsterdam, The Netherlands; Innsbruck Medical University, Austria

## Abstract

The implementation of expanded newborn screening programs reduced mortality and morbidity in medium-chain acyl-CoA dehydrogenase deficiency (MCADD) caused by mutations in the *ACADM* gene. However, the disease is still potentially fatal. Missense induced MCADD is a protein misfolding disease with a molecular loss-of-function phenotype. Here we established a comprehensive experimental setup to analyze the structural consequences of eight *ACADM* missense mutations (p.Ala52Val, p.Tyr67His, p.Tyr158His, p.Arg206Cys, p.Asp266Gly, p.Lys329Glu, p.Arg334Lys, p.Arg413Ser) identified after newborn screening and linked the corresponding protein misfolding phenotype to the site of side-chain replacement with respect to the domain. With fever being the crucial risk factor for metabolic decompensation of patients with MCADD, special emphasis was put on the analysis of structural and functional derangements related to thermal stress. Based on protein conformation, thermal stability and kinetic stability, the molecular phenotype in MCADD depends on the structural region that is affected by missense-induced conformational changes with the central β-domain being particularly prone to structural derangement and destabilization. Since systematic classification of conformational derangements induced by *ACADM* mutations may be a helpful tool in assessing the clinical risk of patients, we scored the misfolding phenotype of the variants in comparison to p.Lys329Glu (K304E), the classical severe mutation, and p.Tyr67His (Y42H), discussed to be mild. Experiments assessing the impact of thermal stress revealed that mutations in the *ACADM* gene lower the temperature threshold at which MCAD loss-of-function occurs. Consequently, increased temperature as it occurs during intercurrent infections, significantly increases the risk of further conformational derangement and loss of function of the MCAD enzyme explaining the life-threatening clinical courses observed during fever episodes. Early and aggressive antipyretic treatment thus may be life-saving in patients suffering from MCADD.

## Introduction

Medium-chain acyl-CoA dehydrogenase deficiency (MCADD; MIM #201450) is the most common fatty acid disorder arising from mutations in the *ACADM* gene (MIM #607008). In the absence of catabolic stress individuals with MCADD are mostly asymptomatic, but intercurrent illness or prolonged fasting may lead to episodes of coma with hypoketotic hypoglycemia and hepatic dysfunction [1], [2]. In the absence of newborn screening (NBS) for MCADD, premature death or serious disability occurs in 20% to 25% of children with the disorder [3]. If MCADD is identified by NBS, families are instructed to avoid prolonged fasting and to have their children hospitalized for glucose infusion during acute illness. These measures reduce the frequency of sudden death in MCADD, but unfortunately, some patients die in spite of early diagnosis and implementation of emergency procedures [4]. Still occurring fatal courses may be due to the incomplete understanding of the underlying molecular phenotype linked to the mutations found in these patients.

Eighty percent of symptomatic patients diagnosed before the establishment of expanded NBS programs in Europe and in the US are homozygous for the mutation p.Lys329Glu (c.985A>G), 18% carry this allelic variant in the heterozygous state [5], [6] with an allele frequency of 90% in this group. Thus, this mutation is tightly linked to the risk of clinical disease manifestation. MCADD patients identified by expanded NBS show a considerably wider genetic heterogeneity with the p.Lys329Glu mutation present only on 47% to 63% of defective alleles [3], [7]–[10]. p.Tyr67His (c.199C>T) is the second most prevalent mutation identified in newborns with MCADD and is discussed to be less severe [7], [9]–[12]. In addition, numerous novel mutations have been identified in the NBS cohort, leading to the current number of 81 different *ACADM* mutations with 80% of these being missense or small insertions/deletions (www.hgmd.org). The clinical relevance of the majority of these mutations is so far unknown. Notably, novel genotypes identified in NBS are associated with risk of sudden death [4]. Clinical observation may support the hypothesis that the two most common mutations in the *ACADM* gene causing the amino acid substitutions p.Lys329Glu, the predominant variant in symptomatic patients, and p.Tyr67His, a biochemically mild variant solely found in presymptomatic infants in NBS, form the two ends of a phenotypic spectrum in MCADD.

Experimental evidence led to the current view that missense induced MCADD is a protein misfolding disease with a loss-of-function molecular phenotype [13]–[16]. In spite of differences in severity both p.Lys329Glu and p.Tyr67His caused protein misfolding [16], [17]. In a previous study, we provided first experimental evidence for the impact of eight additional *ACADM* mutations identified in NBS on MCAD structure and function and all of these were clearly associated with conformational derangement and decreased protein stability [16]. High rates of aggregation and degradation upon recombinant expression indicated a severe protein misfolding molecular phenotype for two further mutations from Bavarian NBS, namely p.Asp223Gly and p.Ile331Thr, and precluded these variants from further experimental exploration [16].

The analysis of the structural consequences of side-chain replacements in the MCAD protein may contribute to a better understanding of the mechanisms underlying mutation-induced protein misfolding and loss of enzyme function. MCAD is a homotetrameric protein with each subunit consisting of three structural domains, the N-terminal α-domain (αD_N_), the middle β-domain (βD), and the C-terminal α-domain (αD_C_). Within the family of fatty acid acyl-CoA dehydrogenases the MCAD enzyme shows a comparatively broad spectrum of substrate utilization [18] with the highest affinity for octanoyl-CoA, and contains a FAD moiety as catalytic redox factor. Substrate and cofactor bind to neighboring but structurally distinct grooves. Substrate binding is confined to the individual subunits, whereas parts of the cofactor molecules adopt an inter-subunit orientation.

In an attempt to classify novel mutations found in NBS we established a comprehensive experimental setup which includes circular dichroism (CD) spectroscopy, differential scanning fluorimetry (DSF) monitoring 8-anilino-1-naphtalenesulfonic acid (ANS) and flavin adenine dinucleotide (FAD) fluorescence, right angle light scattering, and kinetics of thermal inactivation. This allowed for detailed analysis of the conformation of wild-type and variant MCAD proteins focusing on a domain-related view of folding and mutation-induced unfolding events in the MCAD protein. With fever being a distinctive clinical risk factor for patients with MCADD, special emphasis was put on structural and functional derangements related to thermal stress. Moreover, we summarized and quantified the structural data and visualized the molecular protein misfolding phenotype of the variant MCAD proteins in a 3D plot comparing them to the wild-type, to p.Lys329Glu, the classical severe mutation, and to p.Tyr67His, discussed to be a mild mutation.

## Materials and Methods

### Mutations

In a previous study [16] we analyzed oligomerization, enzyme kinetics, and overall stability of the MCAD wild-type protein and 10 variant MCAD proteins derived from *ACADM* missense mutations identified in Bavarian NBS. In this study, we provide detailed conformational analysis of wild-type and eight variant MCAD proteins, p.Ala52Val, p.Tyr67His, p.Tyr158His, p.Arg206Cys, p.Asp266Gly, p.Lys329Glu, p.Arg334Lys, and p.Arg413Ser that were amenable to recombinant expression and purification. Mutations were grouped according to the localization of the respective amino acid side-chain replacement within the three structural domains of the MCAD protein: i) N-terminal α-domain (αD_N_), p.Ala52Val (c.155C>T), p.Tyr67His (c.199T>C), ii) middle β-domain (βD), p.Tyr158His (c.472T>C), p.Arg206Cys (c.616C>T), p.Asp266Gly (c.797A>G), and iii) C-terminal α-domain (αD_C_), p.Arg334Lys (c.1001G>A), p.Arg413Ser (c.1237C>A). A color code linked to the individual protein domains (green, αD_N_; blue, βD; red, αD_C_) was used throughout. Mutations are summarized in Table 1.

**Table 1:** Mutations.

cDNA a	Precursor protein	(Trivial name)	Mature protein c	(Trivial name)
c.155C>T	p.Ala52Val	A52V	p.Ala27Val	A27V
c.199T>C	p.Tyr67His	Y67H	p.Tyr42His	Y42H
c.472T>C	p.Tyr158His	Y158H	p.Tyr133His	Y133H
c.616C>T	p.Arg206Cys	R206C	p.Arg181Cys	R181C
c.797A>G	p.Asp266Gly	D266G	p.Asp241Gly	D241G
c.985A>G	p.Lys329Glu	K329E	p.Lys304Glu	K304E
c.1001G>A	p.Arg334Lys	R334K	p.Arg309Lys	R309K
c.1237C>C	p.Arg413Ser	R413S	p.Arg388Ser	R388S

a Reference sequence: GenBank accession no. M16827.1. Nucleotide numbering starts with A of the ATG initiation codon as +1.

c After cleavage of the 25 amino acid N-terminal mitochondrial targeting sequence.

### Nomenclature of Sequence Variations

GeneBank NM_000016.4 was used as cDNA reference sequence. The recommendations of the Nomenclature Working Group [19] were followed. Nucleotide +1 is the A of the ATG-translation initiation codon. Amino acid numbering starts with the translation initiator methionine as +1. Additionally, the traditional nomenclature system of the MCAD protein is shown in a separate column in Table 1. In this system, amino acid numbering starts at codon 26 with the first 25 amino acids forming a mitochondrial targeting peptide and being cleaved off to produce the mature MCAD monomer [20].

### Plasmid construction

The cDNA of the human *ACADM* gene (*ACADM*-encoding pKK223 plasmid obtained as a gift from Jerry Vockley, Pittsburgh, USA) was cloned into the prokaryotic expression vector pMALc2X (NEB) encoding an N-terminal MBP (maltose-binding protein) and a Factor Xa cleavage site. *ACADM* mutations were constructed by use of the QuikChange Site-Directed Mutagenesis Kit (Stratagene) and verified by DNA sequencing.

### Transient expression of wild-type and variant MCAD in COS-7 cells

COS-7 cells were maintained in RPMI 1640 medium with stable glutamine supplemented with 10% fetal bovine serum and 1% antibiotics (PAA). For transient expression of wild-type and variant MCAD proteins, wild-type and mutant *ACADM* cDNA was subcloned in pEF-DEST51. After transfection of 2 million cells with 2 μg DNA using the Amaxa electroporation system (Lonza), cells were cultured for 48 h in basic medium. Culture medium was changed every 24 h. Cells were harvested by scraping and lysed by three freeze–thaw cycles in a lysis buffer (20 mM Hepes pH 7.0, 200 mM NaCl containing 1% Triton X-100, 100 μg/ml digitonin and proteinase inhibitors), followed by 20 min centrifugation at 14,000 rpm at 4°C. Supernatants were kept at -80°C until being used for the activity assay.

### MCAD enzyme activity assay

The MCAD enzyme activity was determined essentially as described elsewhere [21] using a reaction medium with the following composition: 200 mM Tris-HCL, 0.2 mM phenylpropionyl-CoA, 1 mM ferrocenium hexafluorophosphate, pH 8.0. Final protein concentration in the assay was 0.02 mg protein/ml. Separation of substrate and product was performed by UHPLC-UV.

### Prokaryotic expression and purification

Expression and purification were essentially performed as previously described [16]. In brief, the MCAD wild-type pMALc2X expression plasmid was used to transform the *E. coli* strain BL21-CodonPlus (Stratagene), whereas the *E. coli* strain DH5α (Invitrogen) was co-transformed with expression plasmids of variant human *ACADM* and *pGroESL* [22]. Bacteria transformed with wild-type MCAD were grown in 2 l of dYT medium supplemented with 100 μg/ml ampicillin, while variant proteins were grown in the presence of 100 μg/ml ampicillin and 50 μg/ml chloramphenicol. Protein overexpression was induced with 0.01 mM isopropylthio-β-D-galactoside (IPTG) at a post induction temperature of 28 °C for 20 hours. Recombinant proteins were purified at 4 °C using ÄKTApurifier and ÄKTAprime systems (GE Healthcare). After loading the crude extract on a MBPTrap affinity chromatography column (GE Healthcare), equilibrated with column buffer (20 mM Tris-HCl pH 7.4, 200 mM NaCl, 1 mM EDTA, 1 mM DTT), unbound proteins were washed out and MBP fusion proteins were eluted with the same buffer supplemented with 10 mM maltose, followed by size-exclusion chromatography using a HiLoad 16/600 Superdex 200 column (GE Healthcare) equilibrated with size-exclusion buffer (20 mM HEPES pH 7.0, 200 mM NaCl). The isolated tetrameric fusion proteins were pooled and incubated with Factor Xa (Novagen) for 12 hours at 4 °C to cleave off the MBP tag using a protease to protein ratio of 1∶20 (U μg) followed by final re-chromatography (HiLoad 16/600 Superdex 200 column, GE Healthcare) in size-exclusion buffer. The concentration of the isolated tetrameric fraction was determined using the fluorescent dye binding Quant-iT assay (Invitrogen). All purification steps of wild-type MCAD are shown in Figure S1A and wild-type and variant MCAD proteins were purified to homogeneity (Figure S1B).

### Circular dichroism spectroscopy

Far-UV CD spectra were recorded using a Jasco J-715 spectropolarimeter (Jasco, Gross-Umstadt, Germany) equipped with a PTC 343 Peltier element. Experiments were performed in quartz cuvettes with 0.1 cm path length at a protein concentration of 0.15 μg/μl and far-UV spectra were recorded from 190 to 260 nm in 10 mM potassium phosphate, pH 8.0 at 20 °C. 16 spectra were accumulated for each protein analyzed and subsequently baseline corrected. The composition of secondary structure elements was calculated using the CD spectra deconvolution software CDNN [23]. Thermal transition experiments were monitored at 208 nm with a heating rate of 20 °C per hour. Data were normalized by setting the signal of the native protein to 1.

### ANS and FAD differential scanning fluorimetry

Fluorescence measurements with the wild-type and variant MCAD enzymes (0.5 μg/μl) diluted in 20 mM HEPES buffer, pH 7.0 and 200 mM NaCl were performed on a Cary Eclipse fluorescence spectrophotometer equipped with a temperature-controlled Peltier multicell holder (Varian). Intrinsic FAD fluorescence and binding of the hydrophobic dye 8-anilino-1-naphtalenesulfonic acid (ANS, Sigma-Aldrich) to surface exposed hydrophobic groups was co-monitored (multiwavelength program) during heat-induced denaturation experiments in the temperature range from 25 °C to 65 °C at a heating rate of 1 °C/min. Changes in ANS fluorescence emission were detected at 450 nm (excitation at 395 nm, 5.0/10.0 nm slit widths), whereas intrinsic FAD emission was monitored simultaneously at 530 nm (excitation at 450 nm, 5.0/10.0 nm slit widths). We ensured that no fluorescence resonance energy transfer occurred at the wavelengths used. Transition midpoints (T_m1/2_) were determined graphically with T_m1/2_ being the temperature at which half denaturation occurred. Significances between wild-type and variant proteins were calculated by one-way ANOVA followed by a Dunnett’s post test (GraphPad Prism 5.0).

### Right angle light scattering

Right angle light scattering (RALS) experiments were performed on a Cary Eclipse fluorescence spectrophotometer equipped with a temperature-controlled Peltier multicell holder (Varian). Samples contained 0.08 μg/μl protein diluted in 20 mM HEPES buffer, pH 7.0 and 200 mM NaCl. The increase in turbidity was monitored in the temperature range from 25 °C to 65 °C at a heating rate of 1 °C/min (excitation at 330 nm, emission 335 nm; 5.0 nm slit widths). Turbidity curves were illustrated with GraphPad Prism 5.0.

### Kinetics of thermal inactivation

Thermal inactivation experiments were performed using a ferrocenium-based spectrophotometric assay [24]. MCAD protein diluted in 100 mM HEPES, pH 7.6 supplemented with 0.1 mM EDTA was incubated at five different temperatures in the range from 32 °C to 44 °C for the indicated time intervals. MCAD enzyme activity was subsequently measured by addition of 10 μM octanoyl-CoA. Residual enzyme activities of *n* = 5 to *n* = 7 independent experiments were normalized to initial enzyme activity prior to incubation (25 °C). The kinetic constants of inactivation *k* were calculated by plotting the residual activity against the measurement time.

Arrhenius plots result from plotting the logarithm of the five kinetic constants against the respective inverse temperature. Activation energy (E_A_) was determined from the slope of the Arrhenius plot and is given as single value without measures of variation.

### 3D plot of structural alterations in variant MCAD proteins

To visualize results from experiments investigating thermal stability, kinetic stability, and conformation of MCAD wild-type and variants, we combined data and developed relative scores assessing the severity of mutation-induced changes. The parameter thermal stability (y-axis) reflects the thermal unfolding behavior. Values were based on the means of transition midpoints of thermal denaturation as determined by CD spectroscopy, ANS-DSF or FAD-DSF. The parameter kinetic stability (x-axis) represents the activation energy (E_A_) as a measure of the threshold from the active to the non-active protein upon application of thermal stress. The parameter conformation (z-axis) arises from the results of CD spectroscopy, ANS-fluorescence or intrinsic FAD-fluorescence reflecting secondary structure, tertiary structure, and structural integrity with no stress applied, i. e. at room temperature. A numerical score was assigned to each of the experimental data with values ranging from 9 (native state) to 1 (non-native state).

## Results

### Structural localization and functional impact of MCAD side chain replacements

**Figure 1:**
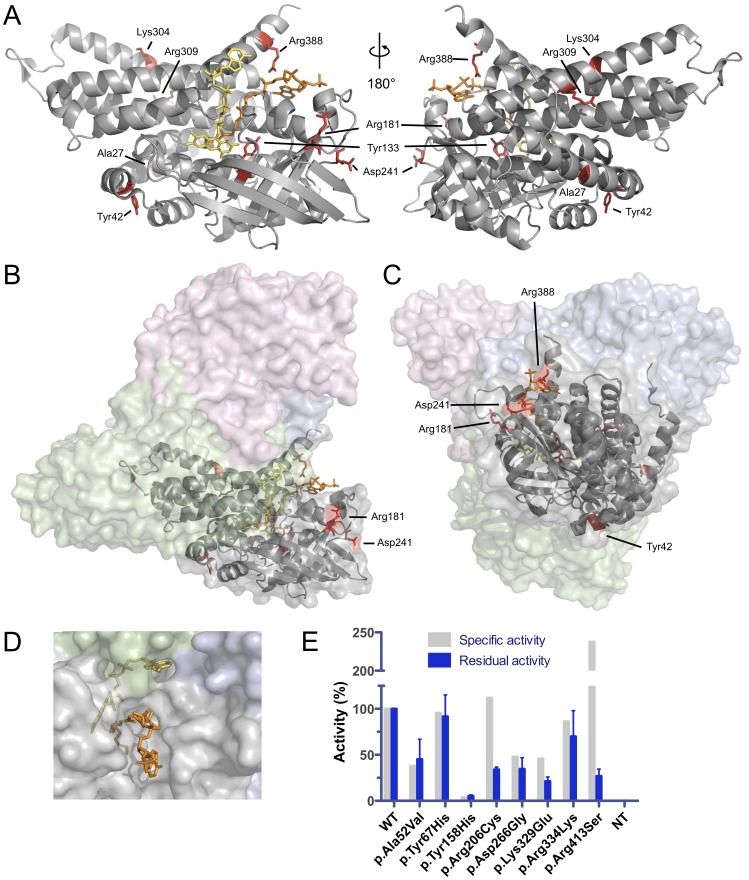
Localization of side chains affected by mutations and residual enzyme function of MCAD variants. (A) Mutations in the *ACADM* gene map to side chains (red) of all regions of the MCAD monomer. The mature MCAD protein is shown and numbering of side chains refers to the primary sequence after cleavage of an N-terminal 25 amino acids mitochondrial targeting peptide (see Table 1). Side chains, FAD cofactor (yellow) and the substrate analogue 3-thiaoctanoyl-CoA (orange) are shown as stick models. (B, C) Structural localization of affected side-chains at the surface of subunit A (gray) relative to the other subunits (B, green; C pink; D, blue) in the MCAD tetramer. (D) Surface view of the MCAD tetramer with 3-thiaoctanoyl-CoA (orange) bound to the substrate binding site and the positioning of FAD (yellow) relative to subunits A (gray), B (green), and D (blue). (E) Relative enzyme activity of wild-type and variant MCAD proteins. Specific activity was determined using purified recombinant protein [16] and residual activity was measured upon expression in COS-7 cells. Data are given as means and SD of three replicates.

The mutations analyzed in this study affect side chains distributed over the entire MCAD protein. Side chains Ala27 and Tyr133 of the mature peptide lacking the 25 amino acid signal sequence are located at the core of the MCAD monomer, Lys304 and Arg309 are positioned at the subunit interface inside the tetramer (Figure 1A), whereas all other side chains (Tyr42, Arg181, Asp241, Arg388) are surface-exposed (Figure 1B and C). Both substrate and cofactor are oriented towards the catalytic core, however, they differentially interact with the MCAD protein (Figure 1D). Only the acyl-moiety of the substrate is buried in the substrate-binding cleft while the coenzyme-A moiety remains solvent-exposed allowing for rapid exchange within catalytic cycles. By contrast, the FAD cofactor is integral to the MCAD 3D structure and diffusion is impeded in the natively folded tetrameric state.

Residual activity of all variant MCAD proteins expressed in COS-7 cells was determined in order to analyze loss of function in living cells. The variants p.Ala52Val, p.Tyr67His, and p.Arg334Lys revealed high residual activity in the range from 45 to 91% as compared to the wild-type, whereas p.Arg206Cys, p.Asp266Gly, and p.Arg413Ser showed activities between 21 and 34%. Only p.Tyr158His displayed low activity of 5%. When compared to the specific enzyme activity of recombinantly expressed and purified MCAD variants (Maier et al 2009), p.Arg206Cys and p.Lys329Glu displayed a significant loss of protein function in the eukaryotic environment. Note that p.Arg413Ser is a kinetic variant with a substrate affinity reduced by two orders of magnitude resulting in a catalytic efficiency (*V*_max_/*K*_m_) of 1.7 compared to that of the wild-type of ∼ 100.

### Variant MCAD proteins show increased hydrophobicity and decreased FAD binding capacity

**Figure 2:**
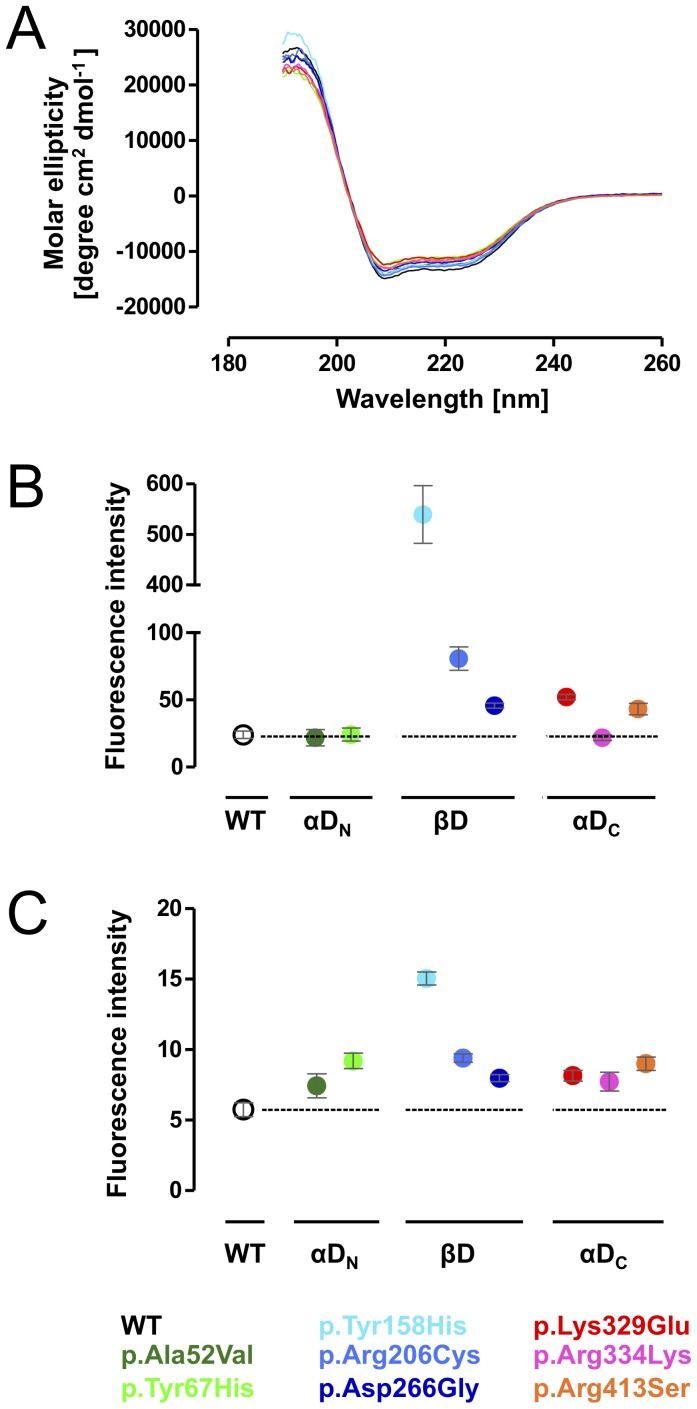
Missense mutations induce conformational alterations of variant MCAD proteins. (A) The composition of secondary structure elements of wild-type and variant MCAD in the absence of thermal stress was determined by circular dichroism applying the CD spectra deconvolution software CDNN [23]. The spectra showed typical secondary structure characteristics of proteins with predominating α-helical content. (B) Hydrophobicity of wild-type and variant MCAD proteins in the absence of thermal stress was determined using the fluorescent dye ANS. An increase in the fluorescence signal points to increased binding of the ANS dye to hydrophobic groups. Data represent means and SEM of three independent experiments. (C) Structural integrity of wild-type and variant MCAD proteins in the absence of thermal stress was determined by intrinsic FAD fluorescence. An increase in the fluorescence signal points to a release of FAD from the MCAD protein. Data represent means and SEM of three independent experiments. (B, C) Data are grouped according to the domain affected by mutations (αD_N_, N-terminal α-helical domain; βD, β-sheet domain; αD_C_, C-terminal α-helical domain).

First, we analyzed missense mutation-induced alterations of variant MCAD conformations with respect to secondary structure, hydrophobicity, and FAD binding in comparison to the wild-type. The relative composition of secondary structure elements was monitored by CD scans recorded in far-UV light between 190 and 260 nm. The CD spectra showed typical secondary structure characteristics of proteins with predominating α-helical structures and a lower amount of β-sheets represented by minima at 208 nm and 222 nm, and a maximum at 193 nm, respectively (Figure 2A). When compared to the wild-type, spectra of variant MCAD proteins showed only slight differences. This was confirmed by quantitative analysis of secondary structure elements by means of CD spectra deconvolution (Table 2). For p.Tyr67His and p.Lys329Glu, a decrease in the relative amount of α-helical structures resulted in a higher share of β-strands. Although to a lesser extent, the two other mutations mapping to αD_C_ (p.Arg334Lys, p.Arg413Ser) showed the same phenomenon, whereas mutations in βD did not induce marked changes in the secondary protein structure.

**Table 2:** Secondary structure analysis (CD spectroscopy).

Secondary structure	WT	p.Ala52Val	p.Tyr67His	p.Tyr158His	p.Arg206Cys	p.Asp266Gly	p.Lys329Glu	p.Arg334Lys	p.Arg413Ser
Helix	41	39	34	42	40	38	35	36	36
Antiparallel	2	2	5	2	2	3	4	3	4
Parallel	9	9	9	9	9	9	9	9	9
Beta-Turn	15	15	16	15	15	15	16	16	16
Random Coil	32	32	32	29	32	32	32	32	32
Total Sum	98	97	97	96	98	97	96	97	97

Data are given in %.

Global conformational changes were determined probing the accessibility of a fluorescent dye (ANS) to hydrophobic groups within the protein. The two variants mapping to αD_N_, p.Ala52Val and p.Tyr67His, showed similar ground-state fluorescence at ambient temperature as the wild-type (Figure 2B). By contrast, all mutations affecting βD (p.Tyr158His, p.Arg206Cys, and p.Asp266Gly) displayed elevated ground-state fluorescence indicating partial unfolding at 25 °C. p.Tyr158His showed the most pronounced changes with an increase by 15-fold, while the ground-state fluorescence of p.Arg206Cys and p.Asp266Gly was increased by 2- and 4-fold, respectively. Two of the three mutations mapping to αD_C_, p.Lys329Glu and p.Arg413Ser, produced a 2-fold elevation of the ground-state fluorescence, whereas hydrophobicity of p.Arg334Lys was similar to that of the wild-type.

Intrinsic fluorescence of the FAD cofactor allows for tracking structural integrity of the MCAD protein as FAD is integral to the natively folded tetrameric state. To investigate whether mutations alter FAD binding capacity, we analyzed FAD fluorescence of MCAD variants at 530 nm after 15 min incubation at room temperature. Thermal denaturation assays demonstrated that FAD is released from the protein in the course of the unfolding process (Figure S2). All mutations induced elevated ground-state FAD fluorescence signals as compared to wild-type MCAD indicating partial loss of FAD binding capacity due to alteration of the native state conformation (Figure 2C). The variant p.Tyr158His showed the most pronounced alterations with an increase in FAD fluorescence by 3-fold. Three mutations, each mapping to one of the three protein domains, were associated with an increase in intensity by 2-fold (p.Tyr67His, p.Arg206Cys, p.Arg413Ser) while the impact of the remaining mutations including p.Lys329Glu was less pronounced.

Taken together, all mutations with the exception of p.Ala52Val showed conformational alterations with individual patterns of derangement as to different structural aspects. p.Tyr67His and p.Lys329Glu had a particular impact on the secondary protein structure, partial unfolding probing global hydrophobicity was most pronounced for mutations mapping to βD, and variants of all domains showed disturbed FAD binding.

### Mutations mapping to βD are associated with accelerated heat-induced unfolding

**Figure 3:**
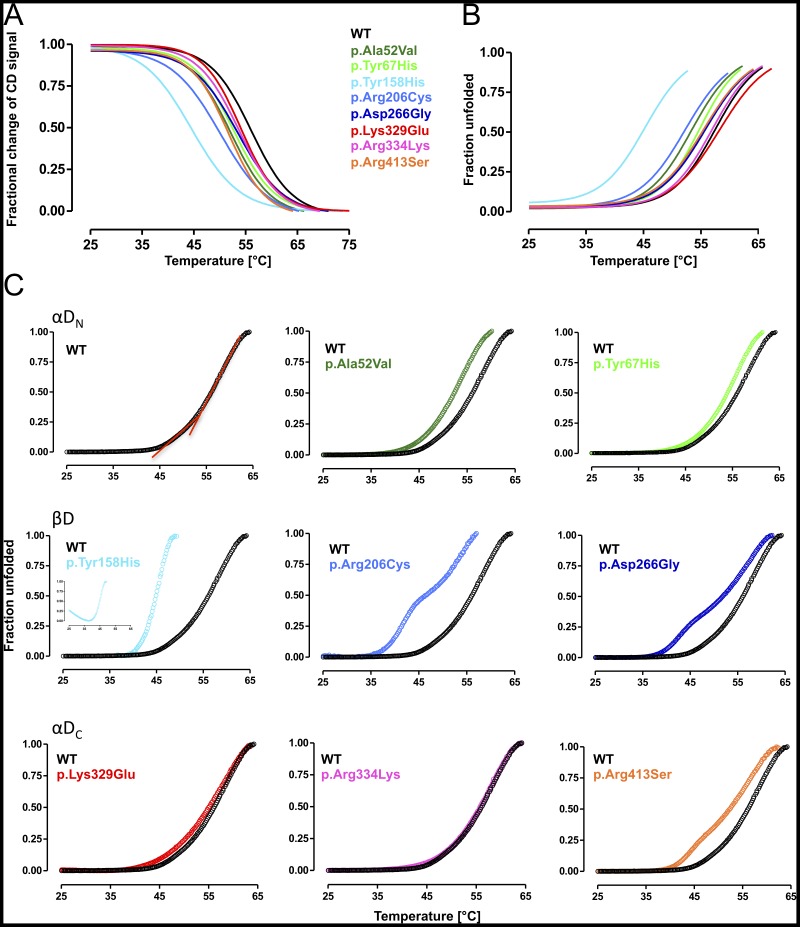
Mutations of the middle β-domain of the MCAD protein are associated with accelerated heat-induced unfolding. (A) Thermal stress-induced denaturation monitored by circular dichroism (CD) probing loss of secondary structure. Denaturation profiles of the fractional change of the α-helical secondary structures of wild-type and variant MCAD were measured at 208 nm. Data are given as non-linear fit of the mean of three independent experiments where *1* represents the native state and *0* the unfolded state. (B) Thermal stress-induced denaturation monitored by FAD differential scanning fluorimetry (DSF) probing FAD binding capacity. The temperature dependent release of intrinsic FAD of wild-type and variant MCAD was measured at 530 nm (excitation 450 nm). Data are given as non-linear fit of the mean of three independent experiments where *0* represents the native state and *1* the unfolded state. (C) Thermal stress-induced denaturation monitored by ANS-DSF probing global unfolding. Unfolding profiles of wild-type and variant MCAD were determined as changes in ANS fluorescence emission at 450 nm (excitation 395 nm). All unfolding patterns revealed a biphasic unfolding mechanism with a distinct low- and high-temperature transition, depicted in two red lines in the unfolding curve of wild-type MCAD. Data are grouped according to the domain affected by mutations (αD_N_, N-terminal α-helical domain; βD, β-sheet domain; αD_C_, C-terminal α-helical domain) and given as mean of three independent experiments where *0* represents the native state and *1* the unfolded state.

Among protein misfolding diseases with a loss-of-function molecular phenotype, the MCADD clinical phenotype has proven to be particularly vulnerable to fever [15], [25], [26]. In order to get structural insight into this disease mechanism, we investigated heat-induced unfolding of variant MCAD proteins by means of CD-spectroscopy, as well as ANS-DSF and FAD-DSF. We monitored denaturation of α-helical secondary structures by means of CD at 208 nm in order to determine transition midpoints between folded and unfolded states (T_m1/2_) (Figure 3A, Table 3). In comparison to wild-type MCAD (T_m1/2_ 56.5 °C), p.Tyr158His was by far the most instable variant with a marked shift of the curve to lower temperatures and a T_m1/2_ deviation of 12 °C (T_m1/2_ 44.5 °C). p.Arg206Cys also displayed markedly reduced stability with a decrease in T_m1/2_ by 6.5 °C. All other variants exhibited a reduced stability of the secondary structure, though to a lesser degree with T_m1/2_-deviations of 2.5 to 4 °C. In this group, p.Arg413Ser and p.Ala52Val showed the most pronounced shift (T_m1/2_ 52.0 °C), the variants p.Tyr67His, p.Arg334Lys, and p.Asp266Gly were in the medium range, whereas p.Lys329Glu was the most stable variant (T_m1/2_ 54.0 °C).

**Table 3:** Mean transition midpoints of thermal denaturation (CD spectroscopy, FAD fluorescence, ANS fluorescence).

	CD a	ANS-DSF^ b^		FAD-DSF^ c^	
	T_m1/2_ (°C)	T_m1/2_ (°C)	SEM	T_m1/2_ (°C)	SEM
WT	56.5	56.4	0.53	58.3	0.53
p.Ala52Val	52.0	52.2	0.37	54.2	0.37
p.Tyr67His	52.5	52.2	0.32	55.7	0.32
p.Tyr158His	44.5	44.9	0.43	46.3	0.43
p.Arg206Cys	50.0	49.1	0.30	52.2	0.30
p.Asp266Gly	53.5	51.4	0.70	56.4	0.70
p.Lys329Glu	54.0	55.1	0.29	59.0	0.29
p.Arg334Lys	53.5	55.5	0.33	57.4	0.33
p.Arg413Ser	52.0	52.1	0.52	56.1	0.52

a Three denaturation profiles were combined to calculate one value for T_m1/2_.

b,c Assayed in triplicates, data are given as means and SEM.

To analyze heat-induced loss of native-state conformation, we monitored FAD fluorescence emission at increasing temperatures. The MCAD wild-type protein showed a T_m1/2_ of 58.3 °C denoting the midpoint of transition from the native cofactor-bound state to the unfolded state with complete cofactor release (Table 3, Figure S2). None of the variants showed marked changes in the shape of the denaturation curves. However, most curves were shifted to lower temperatures indicating early FAD release upon thermal stress (Figure 3B). Surprisingly, the variant p.Lys329Glu was associated with a shift of the FAD fluorescence curve to higher release temperatures (T_m1/2_ 59.0 °C). For p.Arg334Lys we observed a curve virtually mapping to that of wild-type MCAD and p.Asp266Gly, p.Arg413Ser, and p.Tyr67His displayed only moderately left-shifted curves. Three variants (p.Ala52Val, p.Arg206Cys, p.Tyr158His) mapping to αD_N_ and βD were associated with structural rearrangements at the FAD binding site leading to FAD release at lower temperatures. Again, the variant p.Tyr158His showed the most pronounced alteration with a ΔT_m1/2_ of 12 °C (Table 3) correlating well with the shift in T_m1/2_ observed in CD-spectroscopy experiments. Interestingly, the amino acid residue affected by this mutation is an essential part of the active site directly interacting with the FAD cofactor [27].

ANS-DSF is an excellent means to monitor global thermal unfolding events [16], [28]–[30] and transition midpoints for mutations analyzed in this study have been determined before [16]. However, application of varying scan rates as a function of temperature in our previous work did not allow for fine-mapping of unfolding profiles. To enable this, we performed ANS-DSF experiments at a fixed scan rate of 1 °C/min. Under these conditions, the values for T_m1/2_ were well comparable to those obtained by CD-spectroscopy (Table 3). Analysis of the pattern of thermal denaturation curves revealed a subtle biphasic transition with a different slope in the low temperature range between 45 °C and 53 °C for the wild-type (T_m1/2_ 56.2 °C) (Figure 3C). Biphasic unfolding transitions have previously been reported for the MCAD protein [14] and other disease-related multi-domain proteins and different transition phases were associated with unfolding of distinct structural domains [31]–[33].

Variants with an amino acid side-chain substitution in αD_N_ displayed straight curves with a minor shift to lower temperatures indicating moderately decreased stability of the enzyme equally affecting the conformation of all domains. Unfolding patterns of βD variants were severely altered. The p.Tyr158His variant revealed a marked shift in transition and a steeper slope than all other mutations, reflecting the most pronounced destabilization of the protein (T_m1/2_ 44.9 °C). Accordingly, T_m1/2_ was changed to a similar extent as observed by the above methods. p.Arg206Cys and p.Asp266Gly disclosed a marked biphasic transition with a left-shift of the whole curve and an even more pronounced shift of the low-temperature transition. Thus, mutations mapping to βD particularly affected the low-temperature unfolding transition. These observations may indicate that unfolding of βD correlates with the low-temperature transition. Mutations mapping to αD_c_, in particular p.Lys329Glu and p.Arg334Lys, showed unfolding curves very similar to that of the wild-type protein indicating good thermal stability. The p.Arg413Ser variant displayed a reduced mean unfolding transition (T_m1/2_ 51.9 °C) and a clear shift of the low-temperature transition but this was less pronounced than for variants p.Arg206Cys and p.Asp266Gly that also showed biphasic transitions.

In conclusion, considering the localization of side chain replacements in different domains, mutations mapping to βD displayed the most pronounced alterations affecting all parameters tested. In addition to βD variants, p.Ala52Val of αD_N_ was associated with early loss of FAD binding and the αD_C_ variant p.Arg413Ser displayed a marked left-shift of the low-temperature range unfolding transition.

### MCAD variants are prone to aggregation

**Figure 4:**
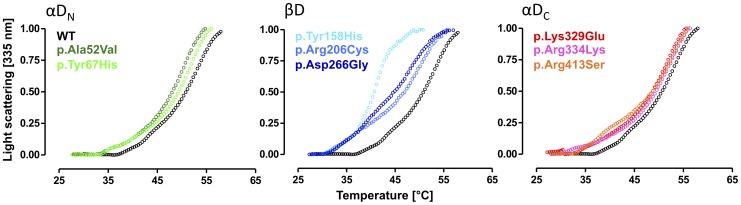
MCAD variants are prone to aggregation. Right angle light scattering experiments probing thermal stress-induced aggregation. The intensity of the scattered light as a function of increasing temperature was determined at 335 nm (excitation 330 nm) for variants arising from mutations mapping to the N-terminal α-domain (αD_N_), variants arising from mutations mapping to the middle β-domain (βD), and variants with amino acid substitutions in the C-terminal α-domain (αD_C_).

To investigate whether thermal stress leads to accelerated protein aggregation of MCAD variants we carried out RALS experiments probing the formation of insoluble aggregates. Based on hydrophobic self-association of the purified proteins, the intensity of the scattered light at 335 nm was detected in right angle to the excitation light of 330 nm and plotted as a function of temperature applied to the sample (Figure 4). Mutations mapping to αD_N_ induced a slight shift of the onset of aggregation to lower temperatures resulting in curves indicative for a similar aggregation behavior as the wild-type protein. Also in this set of experiments MCAD variants of βD displayed the most distinct alterations. Aggregation curves for all three variants (p.Tyr158His, p.Arg206Cys, p.Asp266Gly) were markedly shifted indicating an earlier onset of aggregation than observed for the wild-type and all other variants analyzed here. For p.Tyr158His we observed the most pronounced left-shift with the steepest slope. The pattern of aggregation for mutations mapping to αD_C_ was moderately left-shifted and comparable to that observed in αD_N_.

In summary, missense mutations in the *ACADM* gene mapping to αD_N_ or αD_C_ only showed minor acceleration in the formation of insoluble aggregates upon thermal stress, whereas variants of βD were distinctly aggregation prone and induced aggregation already at lower temperatures.

### Kinetics of thermal inactivation

**Figure 5:**
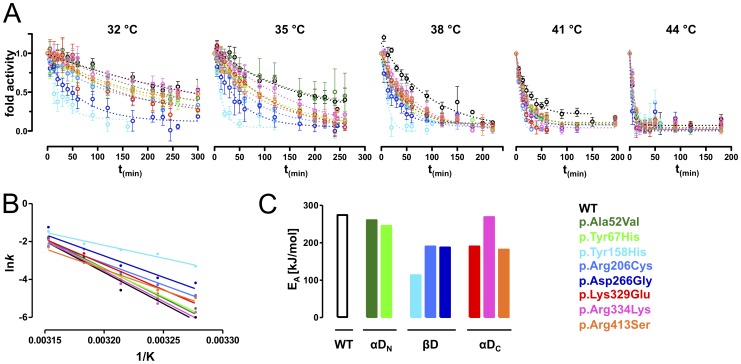
Kinetics of thermal inactivation. (A) Kinetics of thermal inactivation experiments probing dynamics of heat-induced loss of function. Wild-type and variant MCAD proteins were incubated at indicated temperatures and residual activity was plotted as a function of incubation time. (B) Arrhenius Plots resulting from kinetic constants *k* of time-dependent thermal denaturation experiments. (C) Activation energies (E_A_) calculated from Arrhenius Plots reflect the energy barrier between the conformational state with full residual activity and the non-active state after application of thermal stress.

Following the analysis of the impact of increasing temperatures on protein structure we investigated loss of MCAD enzyme function upon thermal stress. Thermal inactivation of recombinant MCAD wild-type and variants was determined over time at temperatures between 32 °C and 44 °C (Figure 5A). Velocity constants (*k*) were calculated from the resulting thermal inactivation profiles and plots of the logarithm of the kinetic constants (ln*k*) against the inverse temperature revealed a linear Arrhenius relationship (Figure 5B). Differences in the slope of Arrhenius plots reflect distinct activation energies (E_A_) for conversion of MCAD variants from an active conformational state into an inactivated and potentially non-native state (Figure 5C). p.Ala52Val and p.Tyr67His of αD_N_ as well as p.Arg334Lys of αD_C_ showed E_A_ at wild-type level. All other variants mapping to βD and αD_C_ displayed reduced kinetic stability. The lowest E_A_ of all variants was observed for p.Tyr158His.

In summary, kinetics of thermal inactivation allowed to gain insight into unfolding dynamics at the active center of the MCAD protein. Variants located in αD_N_ were completely unaffected, possibly due to the remote position to the active site. Mutations mapping to βD and αD_C_ induced decreased kinetic stability as to both global and local unfolding processes. Notably, a side chain replacement remote to the active site structure, p.Arg334Lys, did not alter dynamics of heat-induced unfolding.

### Structural and conformational consequences of mutations in the *ACADM* gene

**Figure 6:**
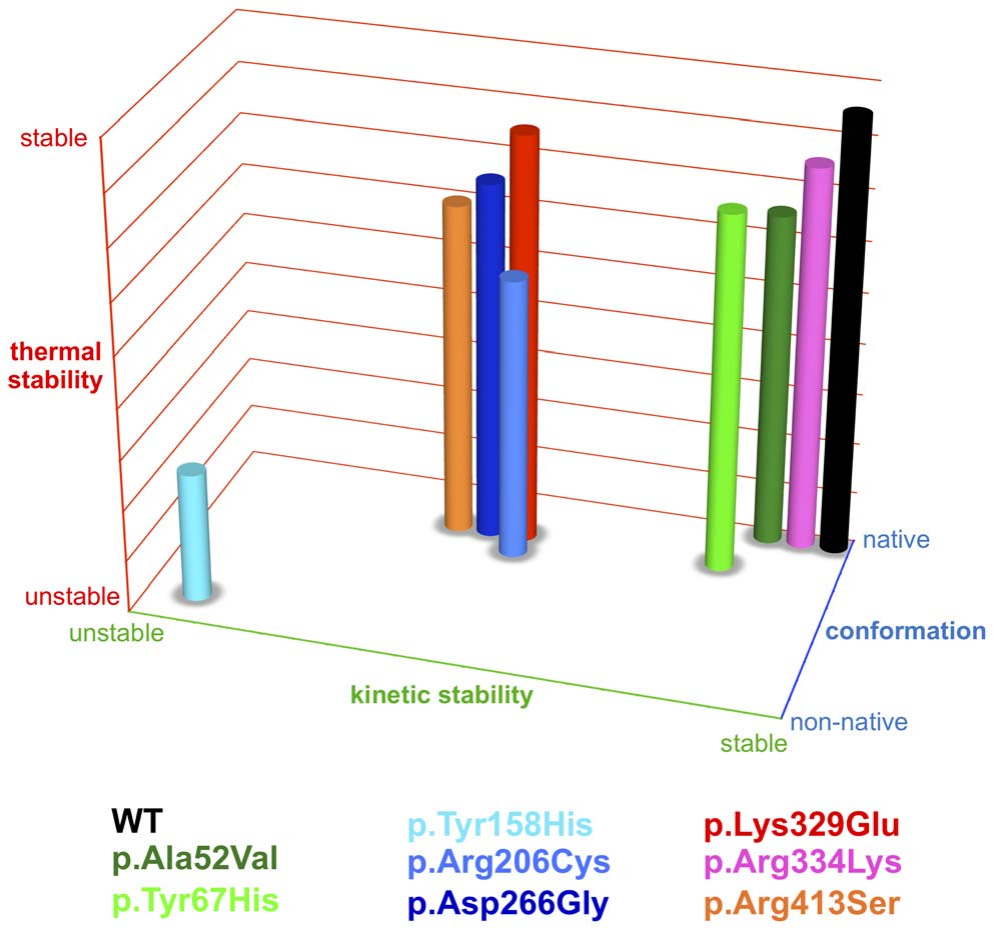
Molecular phenotypes of thermal, kinetic, and conformational stability. Data derived from experiments depicted in Figures 2, 3, and 5 were combined and visualized as 3D plot. Thermal stability (y-axis) refers to midpoints of thermal denaturation determined by CD spectroscopy, FAD-DSF, and ANS-DSF. Kinetic stability (x-axis) refers to the E_A_ determined by thermal inactivation experiments. Conformational stability (z-axis) refers to secondary structure (CD spectra), hydrophobicity (ANS fluorescence), and FAD binding capacity (intrinsic FAD fluorescence) in the absence of heat-induced stress.

To provide an integrated view of the structural consequences of *ACADM* mutations we classified MCAD variants based on conformation, kinetic stability, and thermal stability and visualized the results as a 3D plot (Figure 6).

All mutations analyzed here induced reduced thermal stability when compared to the wild-type. The deleterious structural effects of the p.Tyr158His missense mutation demonstrated by the several experimental approaches applied in this study resulted in opposite positioning of this variant to wild-type in the 3D plot. The other two variants located in the βD, namely p.Arg206Cys and p.Asp266Gly, together with p.Lys329Glu and p.Arg413Ser of αD_C_, built a cluster of relative instability with respect to native-state conformation and thermodynamic stability. The variants p.Ala52Val and p.Arg334Lys were assigned an intermediate position between p.Tyr67His and wild-type with respect to native-state conformation. p.Ala52Val showed a slightly reduced thermal stability and both variants presented with equal or higher thermal stability as compared to p.Tyr67His. Thus, p.Lys329Glu, the classical severe mutation, and p.Tyr67His, discussed to be a particularly mild mutation, did not constitute the edges of the plot.

To summarize, mutations mapping to βD showed the most severe structural alterations with some of the variants displaying more unfavorable structural properties than p.Lys329Glu and others being even less affected than p.Tyr67His.

## Discussion

The introduction of expanded NBS programs including early detection of MCADD raised uncertainties about the clinical relevance of novel *ACADM* mutations identified in individuals showing biochemical abnormalities in the first days of life. Before the screening era p.Lys329Glu was by far the most prevalent mutation, preferentially identified in symptomatic patients presenting with a metabolic crisis [34], [35]. Later, p.Tyr67His was identified as the second most prevalent mutation. To date, it has only been detected in babies identified by NBS and there are no reports of patients presenting clinically with this mutation [7], [11], [15]. On the basis of MCAD enzyme activity determinations, one study came to the conclusion that this variant is of no clinical relevance [36]. The assessment of pathogenicity of mutations identified after positive NBS is of major clinical relevance since the disclosure of a genetic disease is associated with a high burden for the patients and their families [37], [38]. However, data describing the consequences of these mutations at the molecular level and the availability of experimental tools for this assessment, are scarce. At the clinical level, the natural course of the disease in individuals carrying these novel mutations is unlikely to be unraveled as detected asymptomatic babies are protected from metabolic decompensation by the implementation of emergency protocols. At the biochemical level, markers such as blood acylcarnitine concentrations do not correlate well with the risk of metabolic decompensation [39], [40]. A recent study established risk stratification based on residual MCAD enzyme activity obtained from patient blood cells and showed that an enzyme activity of less than 1% is associated with an increased risk of fatal neonatal presentation and enzyme activities below 10% enhance the risk of hypoglycemia [41]. However, reported enzyme activities linked to single mutations vary over a wide range and can differ substantially when residual enzyme activity is analyzed in patient cells or after overexpression of variant proteins [7], [16], [36], [42], [43]. Furthermore, in all cohorts evaluating the pathogenicity of *ACADM* genotypes [11], [36], [42] mutations identified in NBS cohorts are mostly observed in compound heterozygosity with p.Lys329Glu or p.Tyr67His. Only few MCAD variants (5% in [41] and 20% in [11]) were found in other combinations, and even less in the homozygous state.

Prediction tools or 3D-structural analyses are often used to estimate the pathogenic impact of *ACADM* mutations. However, this approach often is not straight-forward and may lead to misassumptions. Based on visual analysis of side-chain replacements in the MCAD 3D structure, one would judge mutations p.Tyr67His, p.Arg206Cys, p.Asp266Gly, and p.Arg413Ser, all affecting protein surface regions, as not damaging. In the case of p.Arg413Ser (mapping to Arg388 in the mature protein) the interaction with the substrate, however, would suggest a loss-of-function phenotype. At the experimental level, we showed that p.Arg413Ser, p.Asp266Gly (Asp241), and p.Arg206Cys (Arg181) displayed a severe structural phenotype, whereas only Tyr67His (Tyr42) showed a mild structural phenotype in accordance with the mild clinical phenotype associated to this mutation. The PolyPhen-2 polymorphism phenotyping tool (www.genetics.bwh.harvard.edu/pph2/) predicts the possible impact of an amino acid substitution on structure and function of a human protein. A query for p.Tyr67His resulted in a score of 0.077 and the side chain replacement was classified as benign. Mutations p.Arg206Cys and p.Arg413Ser were scored 0.999 and classified as probably damaging. However, a score of 0.000 for p.Asp266Gly suggested a benign structural phenotype. This *in silico* prediction did not match our observation of a severe structural phenotype for p.Asp266Gly resembling that of p.Arg206Cys and p.Arg413Ser. Thus, elucidating the molecular consequences of mutations on variant proteins in model systems, which generate data specific to individual MCAD variants, is considered to be a useful tool to gain insight into their pathogenicity.

We previously provided first evidence that *ACADM* mutations identified in newborn screening induce considerable structural alterations [16]. In the work presented here we significantly expanded the experimental program focusing on the effect of thermal stress on conformational integrity as well as on enzymatic function. In a domain-related view, we tracked mutation-induced structural derangements with respect to αD_N_, βD, and αD_C_. A 3D plot summarizing and quantifying the structural phenotype of the variants in comparison to the wild-type on one hand and to the two benchmark mutations p.Tyr67His and p.Lys329Glu on the other, allowed for visualization of the severity of conformational derangement induced by *ACADM* mutations identified by newborn screening. Notably, the mild p.Tyr67His and the severe p.Lys329Glu did not constitute the phenotypic extremes of the panel of mutations analyzed. The classification applied here rather resulted in two groups of variant MCAD proteins. The group associated with mild structural deteriorations comprised the wild-type protein and p.Tyr67His as well as p.Ala52Val and p.Arg334Lys. The other group presenting moderate to severe structural deteriorations comprised p.Lys329Glu as well as p.Arg206Cys, p.Asp266Gly, p.Arg413Ser and at the extreme, p.Tyr158His. Interestingly, mutations identified in NBS either induced structural alterations that were less severe than those observed for p.Tyr67His or more severe than those seen in the presence of p.Lys329Glu. Moreover, mutations p.Tyr67His and p.Lys329Glu displayed similar behavior with respect to various parameters of protein conformation and stability. For both variants only moderate changes were observed with respect to conformation and thermal unfolding or aggregation. Furthermore, both variants displayed a similar shift in the relative distribution of secondary structure elements towards β-strands and β-turns. However, p.Tyr67His and p.Lys329Glu were clearly separated by kinetic stability. This parameter was the best discriminator for both groups. By contrast, thermal stability as determined by DSF assays did not discriminate these two groups.

Based on the data from the present study, all mutations mapping to βD can be categorized as prone to misfolding, unfolding, and functional inactivation. In addition, two out of three mutations affecting residues in αD_C_ can be assigned to the group of severely affected variant MCAD proteins. Variants arising from mutations located in βD displayed more severe structural alterations in all aspects as compared to p.Lys329Glu located in αD_C_. The mutation p.Tyr158His mapping to the core of the MCAD monomer induced the most profound alterations. Mutations p.Arg206Cys, p.Asp266Gly, and p.Arg413Ser showed molecular phenotypes comparable to those induced by p.Lys329Glu and may therefore be classified as just as severe. The latter group of mutations displayed substantial reduction in residual enzyme activity when compared to the respective specific activity, a fact that may underscore the impact of structural derangement on protein function *in vivo*. In the mild group, p.Ala52Val induced alterations comparable to p.Tyr67His and the structural phenotype of p.Arg334Lys was almost unaffected. It seems tempting to assume that the mutations assigned to the mild group or mutations mapping to αD_N_ in general bear a lower risk of metabolic decompensation. Nevertheless, eight mutations mapping to this domain (c.145C>G, Q24E; c.157C>T, R28C; c.233T>C, p.I78T; c.250C>T, p.L84F; c.253G>T, p.Gly85Cys; c.320T>C, p.Leu107Ser; c.346T>G, p.Cys116Gly; c.443G>A, p.Arg148Lys) have previously been associated with intermediate to severe clinical phenotypes including sudden death [7], [11], [44]. Moreover, the mild structural effects determined for p.Ala52Val (Ala27 in the mature protein) are in apparent conflict with the loss in enzymatic function observed. The side chain 27 is not in close proximity to the active site but Ala27 maps to the conjunction of two α-helices and its replacement by valine is likely to displace the far N-termini that exhibit mutual side chain interactions between subunits across the dimer interface. In line with our observation that p.Ala52Val displayed the third lowest melting point of FAD-DSF, proper positioning of N-termini may be of fundamental importance for MCAD structural integrity and consequently for MCAD function. Notably, the classification of the two groups with respect to kinetic stability, *i. e.* thermal stress-induced loss of enzymatic function, correlated with residual activity determined in eukaryotic cells. Residual enzyme activity ranged from 45% to 91% in the mildly affected group and from 5% to 34% in the group of severely affected MCAD variants.

In conclusion, classification of mutations constituting the mild group either as pathogenic or as clinically non-relevant that would exclude patients from clinical follow-up and emergency procedures is still challenging. However, data derived from structural and functional analyses of variant MCAD proteins support the notion that mutations identified in NBS induce significant alterations and may thus be of clinical relevance. In particular, mutations constituting the severe group, p.Tyr158His, p.Arg206Cys, p.Asp266Gly, and p.Arg413Ser may be associated with a significant clinical risk comparable to that associated with the classic *ACADM* mutation p.Lys329Glu. In the absence of adequate biochemical or clinical criteria, the degree of conformational alterations could be a valuable tool to estimate the risk of metabolic decompensation. This may hold particularly true, when data from protein structure analysis are considered in conjunction with functional parameters. On the structural level, the molecular phenotype resulting from *ACADM* mutations identified in NBS presented here depends on the intra-molecular site of structural alterations with the central β-domain being particularly prone to conformational derangement and destabilization. On the functional level it is of critical importance whether and to what extent thermal stress affects the function of the MCAD enzyme.

The clinical observation of metabolic decompensation being considerably aggravated with fever has led to the assumption that the patient’s temperature has impact on the molecular phenotype [4], [7], [10], [14], [39], [41], [45]. We showed here that mutations in the *ACADM* gene lower the temperature threshold at which MCAD loss-of-function occurs. Consequently, increased temperature as it happens during intercurrent infections, significantly enhances the risk of further conformational derangement and loss of function of the MCAD enzyme explaining the life-threatening clinical courses observed during fever episodes in patients suffering from MCADD. Two possible strategies may help to address this issue. First, at short notice, the necessity of early and aggressive antipyretic treatment to avoid further loss in MCAD enzyme activity should be emphasized towards caregivers. Secondly, in the medium term, the development of pharmacological chaperones that bind to the variant MCAD protein and induce its conformational stabilization and functional rescue would provide a specific treatment strategy protecting MCADD patients from metabolic decompensation, morbidity, and early death. This view is strongly supported by positive experiences with two approved drugs acting as pharmacological chaperones in genetic diseases, sapropterin dihydrochloride for phenylketonuria [46], [47] and tafamidis for familial amyloid polyneuropathy [48].

## Supporting Information

Figure S1 **Purification of MCAD wild-type and variant proteins.** (A) Complete representation of all purification steps of wild-type MCAD expressed in *E. coli* BL21-CodonPlus. Lane 1, not transformed BL21-CodonPlus cells as negative control. Lane 2, crude extract of lysed BL21-CodonPlus transformed with MCAD pMAL-c2X. Lane 3, pooled fraction after affinity chromatography and size-exclusion chromatography showing high yield of MBP-MCAD fusion protein and a minor share of MBP protein. Lane 4, equal amounts of MBP protein and MCAD protein after cleavage by factor Xa. Lane 5, pooled tetrameric fraction after re-chromatography by size-exclusion chromatography. (B) Pooled tetrameric fraction of wild-type and variant MCAD proteins after factor Xa cleavage and re-chromatography.
(TIFF)

Figure S2 **Intrinsic fluorescence of cofactor FAD allows for determination of structural integrity of the MCAD protein.** (A) FAD fluorescence scans of the saturated and not saturated MCAD wild-type before (native) and after (denatured) thermal denaturation in comparison to the fluorescence of an FAD standard with the same subunit concentration as the protein sample. (B) Thermal denaturation curves of temperature dependent intrinsic FAD release from MCAD wild-type due to partial or complete unfolding of the enzyme monitored at 530 nm (excitation 450 nm) compared to the fluorescence changes of an FAD standard with the same subunit concentration.
(TIFF)
