# Supplementary figures and images for: The Domain-Specific and Temperature-Dependent Protein Misfolding Phenotype of Variant Medium-Chain acyl-CoA Dehydrogenase
Source: PLoS One. 2014 Apr 9;9(4):e93852. doi: 10.1371/journal.pone.0093852 (PMC3981736; doi:10.1371/journal.pone.0093852)

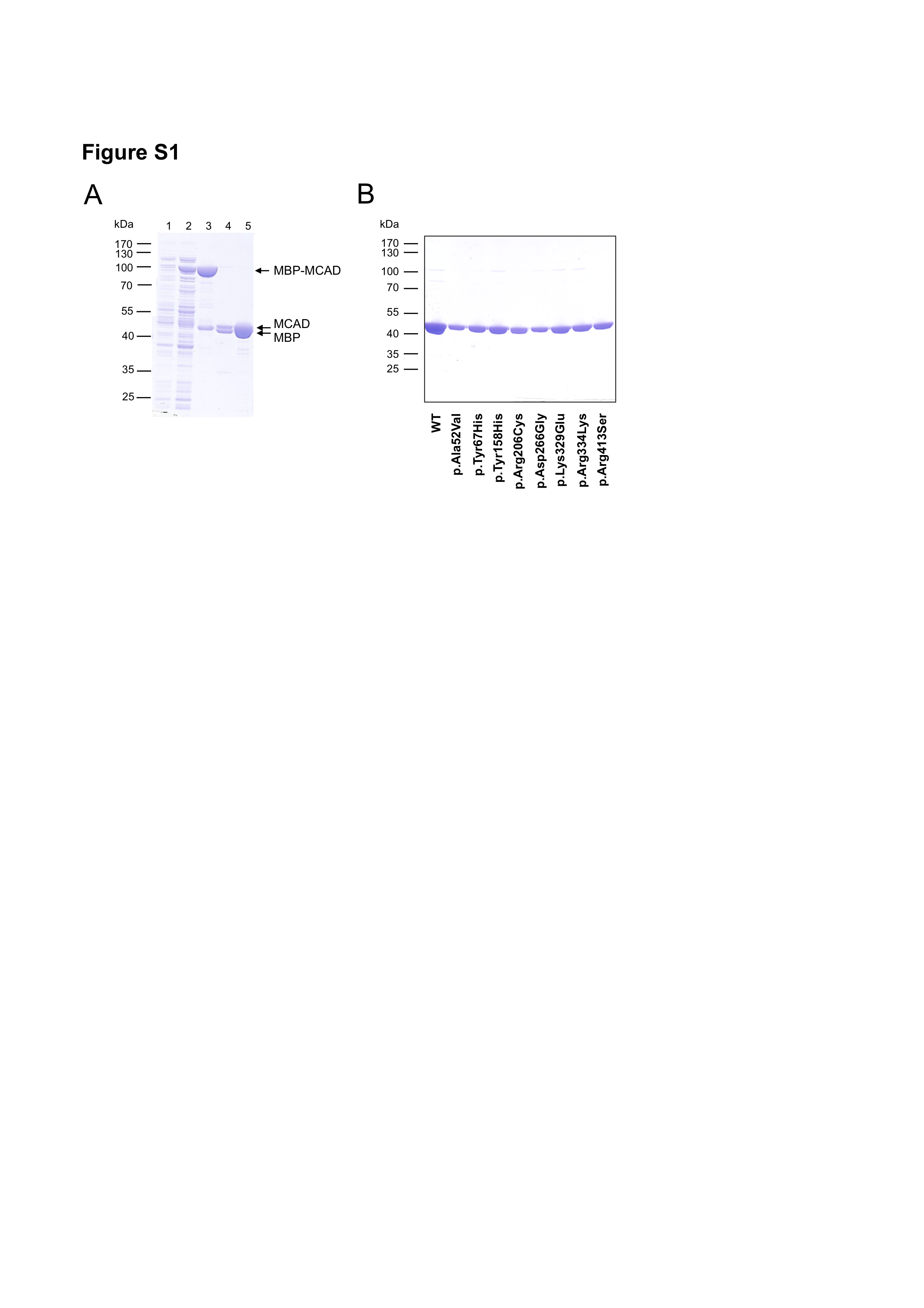

Supplement: Figure S1 — Purification of MCAD wild-type and variant proteins. (A) Complete representation of all purification steps of wild-type MCAD expressed in E. coli BL21-CodonPlus. Lane 1, not transformed BL21-CodonPlus cells as negative control. Lane 2, crude extract of lysed BL21-CodonPlus transformed with MCAD pMAL-c2X. Lane 3, pooled fraction after affinity chromatography and size-exclusion chromatography showing high yield of MBP-MCAD fusion protein and a minor share of MBP protein. Lane 4, equal amounts of MBP protein and MCAD protein after cleavage by factor Xa. Lane 5, pooled tetrameric fraction after re-chromatography by size-exclusion chromatography. (B) Pooled tetrameric fraction of wild-type and variant MCAD proteins after factor Xa cleavage and re-chromatography. (TIFF) [file pone.0093852.s001.tif]

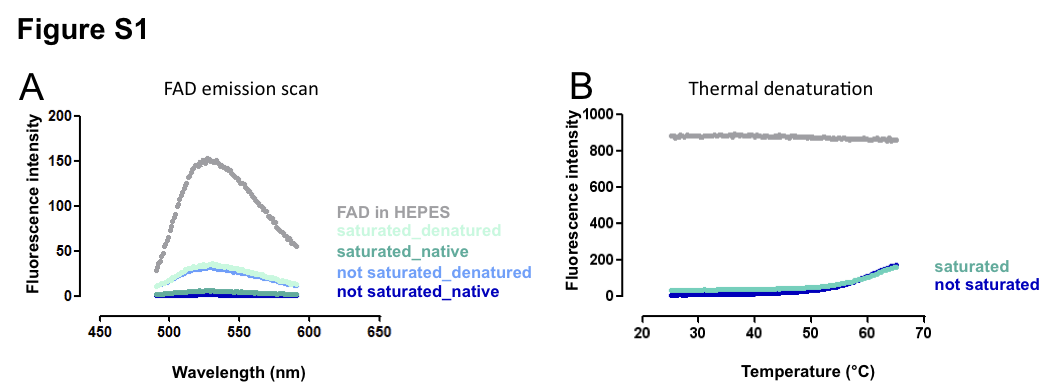

Supplement: Figure S2 — Intrinsic fluorescence of cofactor FAD allows for determination of structural integrity of the MCAD protein. (A) FAD fluorescence scans of the saturated and not saturated MCAD wild-type before (native) and after (denatured) thermal denaturation in comparison to the fluorescence of an FAD standard with the same subunit concentration as the protein sample. (B) Thermal denaturation curves of temperature dependent intrinsic FAD release from MCAD wild-type due to partial or complete unfolding of the enzyme monitored at 530 nm (excitation 450 nm) compared to the fluorescence changes of an FAD standard with the same subunit concentration. (TIFF) [file pone.0093852.s002.tif]
